# The Toxic Effect of Lanthanum on Planaria Is Mediated by a Variety of Ion Channels

**DOI:** 10.3390/toxics6020033

**Published:** 2018-06-18

**Authors:** Wayne Briner

**Affiliations:** College of Health Human Service and Sciences, Ashford University, San Diego, CA 92123, USA; Wayne.Briner@yahoo.com

**Keywords:** lanthanum, planaria, toxicology, calcium, ion channels, potassium, sodium, chloride, magnesium

## Abstract

The effect of toxic concentrations of La^3+^ on the Ca, K, Na, Mg, and Cl channels of planaria was investigated through the use of various agonists and antagonists to those channels. It was demonstrated that La exerts its toxic effects through L-type, but not T-type, Ca channels. La also demonstrated activity at Na, K, Mg, and Cl channels, but, these effects were most likely mediated by other effects of La on Ca activity. However, these interactions appear to be very complex and confounded by oxidative stresses. The study also introduces a planaria stress scale which allows the effects of toxic substances to be examined on a continuum.

## 1. Introduction

Lanthanum and the lanthanide series of metals are being increasingly used in a variety of applications in both industry and medicine. Lanthanides are widely used in the technology industry for the manufacture of materials ranging from semiconductors, optics, and magnets, to experimental superconducting alloys, and experimental therapeutics such as terbium-doped gadolinium nanoparticles as contrast agents [1]. Consequently, there is a risk of increased environmental release of these metals as well as increased human exposure. However, the toxicology and pharmacology of these metals is not completely understood.

Studies that have examined the pharmacology of lanthanum and the lanthanides suggest that they are active voltage-activated calcium channels [2], and lanthanum (La^3+^) has long been used as a calcium channel blocker in pharmacological and physiological studies. Lanthanum, the prototypical lanthanide, is also active at the GABAa receptor chloride channel complex [3] and binds with circulating phosphates, for which it is used extensively in clinical practice. Additional mechanisms of action include alterations of NF-kb signaling, Mg-Ca-ATPase activity, and arachidonic acid metabolism [4,5,6] as well as oxidative damage [7,8]. These reports suggest that the activity of lanthanum is broad and our understanding of the mechanisms of action of the lanthanides is far from comprehensive.

This study seeks to further explore the activity of lanthanum, as the representative lanthanide metal, in planaria. This organism was selected as a model system because of its simple but robust physiology and minimal culture requirements. We also introduce an instrument that allows the effects of toxic substances to be measured with reliability in this organism. We used systematic pharmacologic challenges to La activity to further elucidate the mechanism of La activity in this simple organism, using the constituents of the culture media to guide the investigation.

## 2. Materials and Methods

### 2.1. Husbandry

Planaria (*Girardia dorotocephala*) were initially purchased from Carolina Biological and have since been raised at room temperature in 2-L plastic containers containing dechlorinated tap water and fed frozen beef liver weekly. Worms were fasted for at least 5 days prior to testing.

### 2.2. Test Cultures and Conditions

Unless otherwise specified, all tests were conducted in plastic petri dishes containing 10 mL of culture media. Culture media consisted of a slight modification of Montjuic salts (1.6 mM NaCl, 1 mM CaCl_2_, 1.1 mM MgSO_4_, 0.1 mM KCl, 1.2 mM NaHCO_3_), unless otherwise indicated. Five worms were used in each dish. A minimum of 10 replications were made for each test condition

### 2.3. Test Procedure

Five worms were placed in the plastic culture dish containing 10 mL of culture media and test substances added to the desired concentration. Animal responses to each test condition were evaluated every 24 h using the Planaria Stress Scale (PSS; see Table 1) which was developed in this laboratory to measure the response of planaria to toxics. The average PSS score for each culture dish was used as the unit of measure for analysis. 

The test solution consisted of the drug and metal under consideration dissolved in culture media. All chemicals were reagent grade or better. In some instances the chemicals were insoluble in water and DMSO was used as the vehicle. Probe trials were conducted to ensure that the vehicle had no effect (data not shown). Concentrations of test drugs were the maximum concentration tolerable by planaria as determined by probe trials (data not shown). Concentrations are expressed in mM, unless indicated otherwise. Media pH was checked after the addition of test agents to ensure no change in pH had occurred.

### 2.4. Statistics

Statistical analysis was conducted with one-way ANOVA using SPSS, with *p* < 0.05 indicating statistical significance. An ANOVA was conducted for each of the challenge conditions (calcium, potassium, sodium, chloride, magnesium) with each treatment condition acting as a separate group. A separate ANOVA was conducted for each day. Follow-up statistics were conducted using Tukey’s test with the control condition and La 0.05 mM condition acting as the comparison points for the other treatments. Data is expressed as mean (standard deviation). The effect of a manipulation was considered to be reliable only if a statistically significant effect was seen for at least 3 days.

## 3. Results

### 3.1. Lanthanum Concentration Response Ranging

The PSS is used to measure the stress responses of planaria to toxic substances (see Table 1). It is used as an Excel spreadsheet on a portable device. The number of worms exhibiting the behavior is entered under number of animals and multiplied by the value generating a total for the row and a grand total the divided by the number of animals in the dish. Animals may exhibit more than one characteristic. Dead animals exhibit no other behaviors. For example, for a dish with five animals, three animals exhibiting irregular outlines, two of which exhibited no spontaneous behavior, produces a mean score of 1.6 for the dish. Each dish is reevaluated every 24 h. This instrument may be freely used and modified, so long as proper citation is given.

Test concentrations of LaCl_3_ ranging from 0.01 to 1 mM were conducted with the resulting PSS scores shown in Table 2, which indicates that predictable toxicity begins at a concentration of 0.25 mM. We elected to conduct further experimentation at a LaCl_3_ (simply abbreviated as La for the rest of the paper, for the sake of simplicity) concentration of 0.5 mM, the concentration which resulted in a toxic response that was robust, but, could still be pharmacologically antagonized. Data from control animals (culture media only) are shown for reference in each graph and table and do not represent multiple control trials. Similarly, the La 0.5 mM group acted as a positive control and is used as a reference for all tables, and does not represent multiple La 0.5 mM trials. 

### 3.2. Calcium Challenges

To explore the effect of lanthanum on calcium channels, planaria were exposed to the following test conditions: LaCl_3_ 0.5 mM; nifedipine 0.01 mM; 1-octanol 0.1 mM; CaCl_2_ 6 mM added to the media; LaCl_3_ 0.5 mM and nifedipine 0.01 mM; LaCl_3_ 0.5 mM and 1-octanol 0.1 mM; LaCl_3_ 0.5 mM and CaCl_2_ 6 mM added to the media; calcium-free media; LaCl_3_ 0.5 mM and calcium-free media. Calcium-free media is a modification of the culture media and consisted of: 2.7 mM NaCl; 1.1 mM MgSO_4_; 0.1 mM KCl; 1.2 mM NaHCO_3_.

The results of four days of exposure to each condition are shown in Table 3, with increased calcium levels partially blocking the effect of La and the voltage-activated L-type calcium channel antagonist nifedipine magnifying the toxic effects of La. We consider the effect of Ca-free media on La activity at day three to be spurious; the effect of a manipulation was considered reliable only if there was an effect for at least three days.

### 3.3. Potassium Challenges

To explore the activity of La on potassium channels, planaria were exposed to the following test conditions: LaCl_3_ 0.5 mM; K-free media; KCl 0.1 mM added to the media; minoxidil 2 mM; tetraethylammonium (TEA) 16 mM; quinine HCl 0.05 mM; 4-aminopyridine (4-AP) 0.03 mM; LaCl_3_ 0.5 mM and TEA 16 mM; LaCl_3_ 0.5 mM and 4-AP 0.03 mM; LaCl_3_ 0.5 mM and quinine HCl 0.03 mM; LaCl_3_ 0.5 mM and KCl 0.1 mM added to the media; LaCl_3_ 0.5 and minoxidil 2 mM; LaCl_3_ 0.5 mM and K-free media. K-free media consisted of: 1.7 mM NaCl, 1mM CaCl_2_, 1.1 mM MgSO_4_, 1.2 mM NaHCO_3_.

The results of four days exposure to each condition are shown in Table 4. Potassium-free media completely attenuates the toxicity of La. The K-channel antagonists TEA and 4-AP both accentuate the activity of La. The K-ATP agonist minoxidil appears to have a complex time-dependent effect. The effect of quinine is inconsistent and we consider the significant finding at day one to be spurious; the effect of a manipulation was considered reliable only if there was an effect for at least three days.

### 3.4. Sodium Challenges

To explore the effects of La on sodium channels, planaria were exposed to the following conditions: LaCl_3_ 0.5 mM; Na-free media; carbamazepine 0.2 mM; NaCl 1.6 mM added; lidocaine HCl 0.25 mM; LaCl_3_ 0.5 mM and Na-free media; LaCl_3_ 0.5 mM and carbamazepine 0.2 mM; LaCl_3_ 0.5 mM and NaCl 1.6 mM added; LaCl_3_ 0.5 mM and lidocaine HCl 0.25 mM. Na-free media consisted of: 3.9 mM CaCl_2_, 1.1 mM MgSO_4_, 0.1 mM KCl.

The results for the conditions relevant to sodium are shown in Table 5. Curiously, both enhancing the activity of the Na channel by adding Na to the media, or blocking Na activity with lidocaine, or removing it from the media, enhance the toxicity of La. The effect of carbamazepine on La on day one appears to be spurious; the effect of a manipulation was considered reliable only if there was an effect for at least three days.

### 3.5. Chloride Challenges

The role of Cl channel activity was explored in part by examining the GABAa channel receptor complex. This was accomplished by exposing planaria to the following conditions: LaCl_3_ 0.5 mM; picrotoxin 0.25 mM; GABA 5 mM; Cl-free media; La 0.5 mM and picrotoxin 0.25 mM; LaCl_3_ 0.5 mM and GABA 5 mM; LaCl_3_ 0.5 mM and Cl-free media. Cl-free media consisted of; 1.7 mM NaSO_4_, 1.1 mM MgSO_4_, 1.2 mM NaHCO_3_.

The results of the Cl challenges are presented in Table 6. Removal of Cl from the media consistently attenuated the toxic effect of La. The effects of picrotoxin and GABA are small and inconsistent, the effect of a manipulation was considered reliable only if there was an effect for at least three days.

### 3.6. Magnesium Challenges

The role of magnesium in La activity was explored by exposing planaria to the following conditions: LaCl_3_ 0.5 mM; Mg-free media; MgCl_2_ 1.1 mM added; LaCl_3_ 0.5 mM and Mg-free media; LaCl_3_ 0.5 mM and MgCl_2_ 1.1 mM added. Mg-free media consisted of: 1.6 mM NaCl, 1.0 mM CaCl_2_, 1.1 mM NaSO_4_, 0.1 mM KCl, 1.2 mM NaHCO_3_. The results of manipulating the Mg environment are presented in Table 7. Removing Mg from the media consistently enhanced La toxicity.

## 4. Discussion

That La is active at L-type voltage-activated Ca channels is demonstrated by the synergistic effect of nifedipine to enhance La toxicity. The lack of a synergist effect of La and 1-octanol, a T-type calcium channel antagonist [9], indicates that the T-type calcium channel is not meaningfully involved in La toxicity; although T-type channels are blocked by lanthanide metals [10]. It is interesting to note that 1-octanol is a well-known gap-junction blocker [11], so it would be interesting to investigate the interaction of lanthanides with gap junctions. Yet, that a CaCl_2_ concentration over 6 mM does not completely antagonize the toxicity of La, nor did exposure to Ca-free media, argues that La exhibits its activity through additional mechanisms other than the T- and L-type calcium channels. Another consideration is that at toxic concentrations, La produces oxidative stresses [7,8] which alter Ca signaling, as well as the behavior of a variety of Ca channels [12].

In contrast to the calcium challenges are the findings connected to the potassium challenges. Particularly striking is the finding that K-free media completely and reliably antagonize the effects of La exposure. Yet, 4-aminopyridine (4-AP) and triethanolamine (TEA), both antagonists to voltage-activated K channels, significantly enhanced La toxicity, indicating some linkage to calcium-activated K channels. TEA is a blocker of some calcium-activated potassium channels at submillimolar concentrations [13], but our concentrations were much greater, resulting in a less selective blockade. We postulate that La may bind to the Ca site (Ca bowl) of Ca-activated K channels, enhancing the effects of La. This view is reinforced by the observation that doubling the K levels in the culture media had no effect on La toxicity. The exact role of the various Ca-activated K channels (BK, SK, IK, and others) in La toxicity will require additional study. Minoxidil, a K-channel opener for inwardly rectifying ATP-sensitive K channels, has a biphasic effect, at first attenuating the effects of La, then exacerbating them, suggesting that this family of channels may play a role in the toxicity of La. This is further reinforced by the dramatic effect that the removal of K from the media has. Yet, the addition of more K to the media does not enhance La toxicity, arguing against this. 

Adding yet another layer of complexity to the question of the effects of La on K channels is the role of oxidative stress. La has been shown to produce significant oxidative stress to both the kidney and lung, and presumably other organs [7,8], and oxidative stress has been shown to produce a wide variety of effects on Ca-activated K channels [13]. Further complicating the picture is that La is transported into the cell [14] with unclear effects on Ca channels associated with internal cellular stores, the behaviors of which are also affected by oxidative stress [15].

La uptake by way of the Na/Ca exchanger (NCX) has been demonstrated by Reeves and Condrescu [14], who suggest that La may substitute for Ca in this instance. This explains why adding Na to the culture media enhances La toxicity; more La ends up being transported into the cell. Conversely, once accumulated in the planarial cell, removal of La by the Na-dependent plasma membrane calcium pump (PMCA) may be inhibited by removal of Na from the media, or by the blockage of Na channels by lidocaine. Both of these measures would enhance La toxicity. It is also worth noting that both the PCMA and NCX transporters have been shown to be susceptible to oxidative stress [16].

The addition of Mg, the last cation we investigated, had no effect on La toxicity. However, removal of Mg from the culture media enhanced the toxicity of La. One explanation for this may be the general view that the overall activity of Ca is antagonized by the presence of Mg. However, Mg is also active at the Na/Mg antiporter, the Mg/Ca-activated K channels, and Mg- and voltage-activated Ca channels [13]. The role of Mg at Ca-activated K channels appears to be especially complex [10,17,18]. The blockade of Ca at any of these channels by La can alter a wide variety of cellular functions, and the removal of Mg from the media would enhance that activity even further. La has also been show to replace Mg in a variety of Mg-dependent systems including Ca-ATPase and Mg–Ca transporters [19].

The removal of chloride from the media partially antagonized the effects of La toxicity. However, manipulating the activity of the GABAa chloride channel had no reliable effect on La toxicity. This leads to the reasonable postulation that La has activity at one or more of the Ca-activated Cl channels (CaCCs), although other anion channels may be involved [20,21]. It should also be noted that increasing the Cl concentration by adding KCl and MgCl_2_ to the media (see above) had no effect on La toxicity. It should again be noted that reactive oxygen species also modulate the activity of a variety of anion channels which, relatedly, are also involved in the early stages of apoptosis [22].

This approach does suffer from some shortcomings. Immediate and delayed effects cannot be separated out, and effects on the activity of other molecules, such as enzymes, were not explored. The simultaneous activity of La on several mechanisms at once is also a difficulty. Because the activity of La is indeed very complex, investigators should exercise caution when assigning biological activity to this element.

## 5. Conclusions

In conclusion, caution should be exercised when discussing the effects of La on cell and organismal physiology. Typically thought of as a Ca channel blocker, particularly at the L-type channel, La has direct or Ca-mediated effects on Cl, K, Mg, and Na channels and metabolism. The mechanisms of La activity, especially at toxic concentrations, are quite diverse and complex and will require considerably more study before being completely elucidated. It is also reasonable to anticipate that the activity of other lanthanide metals will be similarly complex.

## Figures and Tables

**Table 1:** Planaria Stress Scale (PSS).

Planaria Stress Scale	Value	# Animals	Total for Row
Dead	15		
Free floating, no contact with container or surface tension	3		
Curled	3		
No response to tactile stimulation	3		
Irregular body outline or head degeneration	2		
Center of dish, not near wall	1		
Lack of spontaneous movement	1		
No signs of stress	0		
Total		Grand Total →	
		Mean →	

**Table 2:** Lanthanum concentration curve data. Planaria response to varying concentrations of La using the PSS; mean (SD). Concentrations are in mM. A La concentration of 0.5 mM gives a robust response, yet, it can still be antagonized. This concentration was used for the remainder of the study.

La	Day 1	2	3	4
Control	0.26 (0.35)	0.34 (0.34)	0.40 (0.31)	0.40 (0.33)
0.06	0.43 (0.42)	0.63 (0.35)	0.53 (0.32)	0.60 (0.28)
0.12	0.98 (0.57)	0.86 (0.32)	1.14 (0.61)	1.04 (0.42)
0.25	3.66 (1.61)	3.79 (2.11)	3.96 (2.48)	3.86 (2.17)
0.5	6.91 (1.49)	7.30 (1.38)	8.00 (1.70)	9.04 (2.06)
1	7.92 (0.87)	7.48 (1.37)	10.16 (1.14)	12.24 (3.35)

**Table 3:** Calcium challenges to La activity. Data are mean (SD) for scores on the Planaria Stress Scale, minimum 10 dishes/cell.

Drug 1	Drug 2	Day 1	Day 2	Day 3	Day 4
La 0.5 ^#-all days^		7.04 (1.58)	7.40 (1.44)	8.05 (1.71)	9.22 (2.18)
La 0.5 ^#-all days^	Nifedipine 0.01	12.95 (2.57) *	14.65 (0.86) *	15 (0) *	15 (0) *
La 0.5 ^#-all days^	Ca-Free	7.18 (1.25)	8.52 (1.16) *	9.14 (1.10)	9.42 (1.50)
La 0.5 ^#-all days^	1-Octanol 0.1	6.62 (1.34)	7.64 (1.07)	8.70 (1.42)	9.00 (2.27)
La 0.5 ^#-all days^	Ca 6	5.38 (1.58)	4.69 (1.37) *	5.29 (1.57) *	6.29 (1.62) *
Control *^-all days^		0.26 (0.35)	0.34 (0.34)	0.40 (0.31)	0.40 (0.33)
1-Octanol 0.1 *^-all days^		0.88 (0.49)	0.90 (0.70)	0.64 (0.44)	0.82 (0.32)
Ca-Free *^-all days^		0.52 (0.31)	0.31 (0.21)	0.49 (0.26)	0.64 (0.45)
Ca 6 *^-all days^		0.51 (0.35)	0.29 (0.37)	0.51 (0.31)	0.46 (0.39)
Nifedipine 0.01 *^-all days^		1.09 (0.63)	0.96 (0.31)	1.00 (0.40)	0.96 (0.19)

* indicates significantly different from La 0.05; ^#^ indicates significantly different from control.

**Table 4:** Potassium challenges to La activity. Data are mean (SD) for scores on the Planaria Stress Scale, minimum 10 dishes/cell.

Drug 1	Drug 2	Day 1	Day 2	Day 3	Day 4
La 0.5 ^#-all days^		7.04 (1.58)	7.04 (1.44)	8.05 (1.71)	9.22 (2.18)
La 0.5 ^#-all days^	TEA 16	8.82 (0.79) *	8.08 (1.38)	10.3 (2.26) *	13.08 (1.35) *
La 0.5 ^#-all days^	4-AP 0.03	8.62 (0.83) *	8.78 (1.82)	10.36 (1.39) *	11.76 (2.03) *
La 0.5 ^#-all days^	Quinine 0.05	8.32 (0.98) *	8.26 (1.73)	9.65 (2.01)	10.58(2.80)
La 0.5 ^#-all days^	K 0.1	7.82 (0.92)	7.60 (1.27)	6.78 (1.40)	9.76 (1.59)
La 0.5 ^#-all days^	Minoxidil 2	4.32 (1.68) *	4.82 (1.64) *	9.18 (2.73)	12.9 (1.09) *
La 0.5	K-Free	0.58 (0.33) *	0.58 (0.42) *	0.42 (0.34) *	0.32 (0.20) *
Control *^-all days^		0.26 (0.35)	0.34 (0.34)	0.40 (0.31)	0.40 (0.33)
Minoxidil 2 *^-all days^		1.25 (0.39)	1.25 (0.31)	1.51 (0.39)	1.58 (0.39)
TEA 16 *^-all days^		1.22 (0.35)	0.72 (0.40)	0.96 (0.27)	0.74 (0.38)
Quinine 0.05 *^-all days^		1.08 (0.48)	0.95 (0.67)	0.83 (0.39)	0.82 (0.20)
4-AP 0.03 *^-all days^		0.51 (0.37)	0.78 (0.31)	0.76 (0.28)	0.80 (0.33)
K-Free *^-all days^		0.47 (0.27)	0.58 (0.31)	0.32 (0.17)	0.50 (0.30)
K 0.1 *^-all days^		0.46 (0.35)	0.36 (0.27)	0.28 (0.27)	0.40 (0.27)

* indicates significantly different from La 0.05; ^#^ indicates significantly different from control. Note that La 0.5 mM and K-free media condition was not significantly different from control.

**Table 5:** Sodium challenges to La activity. Data are mean (SD) for scores on the Planaria Stress Scale, minimum 10 dishes/cell.

Drug 1	Drug 2	Day 1	Day 2	Day 3	Day 4
La 0.5 ^#-all days^		7.04 (1.58)	7.40 (1.44)	8.05 (1.71)	9.22 (2.18)
La 0.5 ^#-all days^	Na-Free	10.15 (1.38) *	12.57 (1.90) *	14.69 (0.51) *	15.00 (0.00) *
La 0.5 ^#-all days^	Carbamazepine 0.2	8.58 (0.77) *	8.62 (0.60)	8.94 (1.15)	10.68 (0.82)
La 0.5 ^#-all days^	Na 1.6	8.34 (0.86) *	9.26 (0.75) *	9.60 (1.45) *	11.70 (0.98) *
La 0.5 ^#-all days^	Lidocaine 0.25	8.28 (0.90) *	9.28 (1.22) *	10.78 (1.40) *	12.82 (0.96) *
Control *^-all days^		0.26 (0.35)	0.34 (0.34)	0.40 (0.31)	0.40 (0.33)
Carbamazepine 0.2 *^-all days^		1.22 (0.42)	1.07 (0.42)	0.85 (0.28)	1.09 (0.22)
Lidocaine 0.25 *^-all days^		1.18 (0.31)	0.82 (0.30)	1.05 (0.26)	0.84 (0.42)
Na 1.6 *^-all days^		0.49 (0.23)	0.38 (0.29)	0.56 (0.35)	0.75 (0.36)
Na-Free *^-all days^		0.36 (0.20)	0.54 (0.30)	0.34 (0.18)	0.54 (0.42)

* indicates significantly different from La 0.05; ^#^ indicates significantly different from control.

**Table 6:** Chloride challenges to La activity. Data are mean (SD) for scores on the Planaria Stress Scale, minimum 10 dishes/cell.

Drug 1	Drug 2	Day 1	Day 2	Day 3	Day 4
La 0.5 ^#-all days^		7.04 (1.58)	7.40 (1.44)	8.05 (1.71)	9.22 (2.18)
La 0.5 ^#-all days^	Picrotoxin 0.25	9.14 (0.30) *	8.04 (0.66)	8.12 (0.81)	8.90 (1.95) *
La 0.5 ^#-all days^	GABA 5	8.30 (0.83) *	8.55 (0.71) *	8.67 (0.97)	10.47 (1.29)
La 0.5	Cl-Free	2.80 (0.63) *^,#^	3.26 (0.28) *^,#^	3.48 (0.87) *^,#^	4.08 (1.47) *^,#^
Control *^-all days^		0.26 (0.35)	0.34 (0.34)	0.40 (0.31)	0.40 (0.33)
Cl-Free *^-all days^		0.46 (0.28)	0.42 (0.21)	0.26 (0.16)	0.36 (0.31)
Picrotoxin 0.25 *^-all days^		0.46 (0.34)	0.42 (0.26)	0.38 (0.21)	0.28 (0.22)
GABA 5 *^-all days^		0.64 (0.32)	0.68 (0.24)	0.84 (0.39)	0.82 (0.37)

* indicates significantly different from La 0.05; ^#^ indicates significantly different from control.

**Table 7:** Magnesium challenges to La activity. Data are mean (SD) for scores on the Planaria Stress Scale, minimum 10 dishes/cell.

Drug 1	Drug 2	Day 1	Day 2	Day 3	Day 4
La 0.5 ^#-all days^		7.04 (1.58)	7.40 (1.44)	8.05 (1.71)	9.22 (2.18)
La 0.5 ^#-all days^	Mg-Free	8.92 (1.02) *	9.24 (2.18) *	12.02 (2.07) *	13.78 (1.44) *
La 0.5 ^#-all days^	Mg 1.1	7.85 (1.17)	7.5 (1.28)	8.42 (1.00)	9.05 (1.72)
Control *^-all days^		0.26 (0.35)	0.34 (0.34)	0.40 (0.31)	0.40 (0.33)
Mg 1.1 *^-all days^		0.82 (0.34)	0.60 (0.28)	0.67 (0.34)	0.56 (0.28)
Mg-Free *^-all days^		0.48 (0.31)	0.40 (0.22)	0.30 (0.27)	0. 40 (0.20)

* indicates significantly different from La 0.05; ^#^ indicates significantly different from control.
